# HPV Vaccine Awareness and Uptake Among Sexually Transmitted Infections Clinic Users: A Cross-Sectional Study in Bologna, Italy

**DOI:** 10.3390/ijerph21111515

**Published:** 2024-11-14

**Authors:** Marta Cleva, Valeria Gaspari, Andrea Ceccarelli, Gabriele Pianese, Davide Griffa, Gionathan Orioni, Christian Cintori, Giuseppe Diegoli, Davide Gori, Marco Montalti

**Affiliations:** 1 Department of Medical and Surgical Sciences, University of Bologna, 40126 Bologna, Italymarco.montalti7@studio.unibo.it (M.M.); 2 Dermatology Unit, IRCCS Azienda Ospedaliero-Universitaria di Bologna, Policlinico S. Orsola Malpighi, 40126 Bologna, Italy; 3 Sector of Collective Prevention and Public Health, Directorate General for Personal Care, Health, and Welfare, Emilia-Romagna Region, 40127 Bologna, Italy; 4 Unit of Hygiene and Medical Statistics, Department of Biomedical and Neuromotor Sciences, University of Bologna, 40126 Bologna, Italy

**Keywords:** HPV vaccine, sexually transmitted infections, sexual and gender minorities, behavioral and social determinants, vaccine uptake, vaccine awareness

## Abstract

Human Papillomavirus (HPV) infection poses a significant health risk, particularly for high-risk groups such as men who have sex with men (MSM), people living with HIV (PLHIV), and transgender individuals. Despite the availability of effective vaccines, uptake among these groups remains suboptimal due to various social and behavioral barriers (BeSD). A cross-sectional survey was conducted at the Sexually Transmitted Infections (STIs) clinic in Bologna, Italy, from 8 April to 12 April 2024 using a paper questionnaire, investigating HPV vaccine uptake and BeSD factors influencing vaccination decisions. Statistical analyses included descriptive statistics and multivariate logistic regression. Among the 236 respondents, PLHIV and transgender individuals demonstrated lower uptake rates (60.0% and 15.6%) if compared to women under 30 years old (72.7%). Concern about HPV infection varied significantly across groups, with MSM showing the highest worry (48.7%). Perceptions of vaccine safety and access were mixed, influencing vaccination decisions. Multivariate analysis indicated that age inversely correlated with infection worry (OR: 0.94, 95% CI: 0.91–0.98), while being a woman under 30 (OR: 164.0, 95% CI: 17.2–1560.18) or MSM (OR: 3.53, 95% CI: 1.37–9.11) was positively associated with vaccine uptake. The study identifies disparities in HPV vaccine uptake among STI clinic users in Bologna, Italy, emphasizing the need for targeted public health campaigns. These campaigns could engage STI clinics and address awareness, safety perceptions, and access barriers to enhance vaccination coverage among sexual and gender minorities.

## 1. Introduction

Human Papillomavirus (HPV) represents one of the most common sexually transmitted infections [1]. Although most of the latter are asymptomatic and transient, persistent infections sustained by “high-risk” HPV types—such as 16 and 18—predispose one to the development of malignant neoplastic lesions affecting the anogenital and oropharyngeal region/trait [2]. Given the infectious etiology, the implementation of a primary preventive strategy based on vaccination is crucial for downsizing and eliminating the risk of HPV-related diseases [3]. Indeed, as estimated by the Centers for Disease Control and Prevention (CDC), up to 92% of HPV-related cancers could be prevented with the HPV vaccine [4].

Nowadays, a vaccination that can prevent infection by nine strains of HPV is available. The HPV vaccine can be given starting at the age of 9 years. Only two doses are needed if the first dose was given before the 15th birthday. Teens and young adults who start the series later, at ages 15 through 26 years, need three doses of HPV vaccine [5].

The vaccination campaign in Italy started in 2008, initially targeting girls from their 12th year of life [6]. However, it was later widened to increase vaccination coverage. Since 2017, vaccination has been provided free of charge to boys from the age of 12 and to other high-risk groups, including individuals living with HIV (PLHIV) and those at increased risk of exposure, such as men who have sex with men (MSM) [7,8]. According to the “Regional Vaccination Prevention Plan (PRPV) 2023–2025” of the Emilia-Romagna Region, sex workers also receive the vaccine free of charge, and recently, it was extended to people undergoing gender transition [9].

Although HPV infection is common among sexually active individuals of all genders, MSM and transgender people, particularly those who are living with HIV, bear a disproportionate burden of HPV infections and related diseases [10,11]. Furthermore, comorbid HPV/HIV infection increases the likelihood of developing HPV-related illnesses [12].

Vaccination data regarding sexual and gender minorities (SGMs) are limited, but the available ones suggest particularly low vaccine completion rates [13,14].

The low uptake could be attributed to several factors and remains an issue of public health concern. Poor knowledge of the importance and effectiveness of vaccination, low perception of the risk of HPV diseases, and barriers for SGMs to access to healthcare due to social stigma and medical mistrust could contribute to the low coverage of the community. Furthermore, communication efforts by health authorities may have been ineffective: historically, vaccination marketing has primarily targeted cisgender women, and currently, the active call is directed only towards girls and boys from the age of 12, neglecting other at-risk categories [15,16,17].

This study aimed to evaluate the uptake of the HPV vaccine among STIs clinic users and target populations in Emilia-Romagna, Italy, and to identify social and behavioral factors associated with non-vaccination. These findings can inform the development of evidence-based strategies and solutions to enhance adherence to the vaccination campaign among these at-risk groups.

## 2. Materials and Methods

### 2.1. Study Design and Participants

A cross-sectional survey study was conducted from 8 April to 12 April 2024, utilizing a paper questionnaire administered at the Sexually Transmitted Infections (STIs) clinic in Bologna, Italy. The STI service, part of the Dermatology Unit, serves as one of the Italian sentinel surveillance centers for the prevention, diagnosis, and treatment of major sexually transmitted diseases. This clinic accommodates approximately 70 patients daily, offering screenings of blood, urine, or biological swabs tailored to the sexual risk and clinical history of each patient.

Upon entering the center, all users were greeted by trained healthcare professionals. The staff distributed the questionnaires, explained the purpose of the study, and helped as needed. The completed questionnaires were collected when users exited the clinic. To ensure that the sample was representative of the center’s user population, the working group administered the questionnaire over a full week during all opening hours.

The inclusion criteria for the study were being 18 years of age or older and having the ability to understand written Italian. Participation in the study was voluntary, and the questionnaire was anonymous. The study was approved by the Bioethics Committee of the University of Bologna (Italy) on 6 February 2024 (protocol number 0031908). The study adhered to the principles outlined in the Declaration of Helsinki. Interventionary studies involving animals or humans and other studies that require ethical approval must list the authority that provided approval and the corresponding ethical approval code. This manuscript adheres to the Strengthening the Reporting of Observational Studies in Epidemiology (STROBE) guidelines for reporting observational research. The checklist provided by STROBE (available at the Strengthening the Reporting of Observational Studies in Epidemiology (STROBE) Statement: Guidelines for Reporting Observational Studies | EQUATOR Network) has been consulted during the preparation of this manuscript to ensure comprehensive and transparent reporting of our observational study. It is included in the Supplementary Materials for reference.

### 2.2. Questionnaire and Data Collection

The questionnaire (see Supplementary Material) comprised nineteen multiple-choice questions and one open question. It was designed to be completed within 5 min and comprised two sections. The first section, consisting of the initial seven questions, pertained to the socio-demographic, educational, gender, and sexual orientation characteristics of the participants. The second section of 13 questions investigated the participants’ health status and specific questions regarding knowledge, perception, and administration of HPV vaccination.

The survey questions were developed based on insights from clinical experience and the collective expertise of a multidisciplinary team of physicians and nurses specializing in this topic. This formulation was informed by analyzing questionnaires previously employed in the published literature. Vaccine-specific sections were structured in alignment with the HPV vaccine survey of the OBVIOUS Project [18] and according to the domains outlined in the WHO BeSD framework [19]: thinking and feeling, social processes, motivation, practical issues, and vaccination.

The questions in both sections featured closed-ended responses or four-point Likert scales.

### 2.3. Statistical Analysis

Numerical variables were reported as mean ± standard deviation (SD), while categorical variables were presented as frequencies and percentages. The determinants of “worry about getting the HPV infection”, “perception of the HPV vaccine as safe”, “perception of the access to get an HPV vaccine as easy”, “Knowledge of vaccine campaign targets in ER”, and HPV vaccine uptake were evaluated using a multivariate analysis. The rating scales of the questionnaires for these domains, ranging from 1 (not at all) to 4 (very), were re-coded into two categories: 1 and 2 for “negative” responses and options 3 and 4 for “positive” responses. Regarding vaccine uptake, in the multivariate analysis, only the responses “yes” and “no” were considered valid, while respondents who did not remember or did not know were excluded. The results of the multivariate analyses were expressed as odds ratios (OR) with standard error (SE) and 95% confidence intervals (CI).

A stepwise regression was performed to determine the variables to be included in the final multiple logistic regression model, based on the results of the univariate models and the principles of parsimony and biological plausibility. All analyses were conducted using Stata software, version 15 (StataCorp, 2017; Stata Statistical Software, Release 15: StataCorp LP, College Station, TX, USA). The significance level was set at 0.05, and pairwise deletion was used for missing data.

## 3. Results

### 3.1. Main Features of the Sample

The study analyzed a sample of 236 users attending the STIs clinic in Bologna, categorized into four HPV vaccine campaign target groups: women under 30 years old (14.0%, n = 33), men who have sex with men (MSM) (33.9%, n = 80), people living with HIV (PLHIV) (6.8%, n = 16), and transgender individuals (3.0%, n = 7). Those not specifically targeted by the HPV vaccine campaign in Emilia-Romagna Region, Italy, were 46.6% (n = 110) of the sample.

The mean age of the participants (n = 196) was 35.0 ± 11.8 years, with PLHIV having the highest mean age (44.6 ± 9.2 years) and women under 30, of course, having the lowest (25.0 ± 2.5 years). Educational levels varied, with the majority holding a high school diploma (34.3%) or a bachelor’s degree (49.1%). Notably, 8.1% of the total sample had only primary or middle school education, and 7.6% had higher education or a PhD.

Financial resources were sufficient for 18.0% of participants to meet their needs “very easily” and for 41.0% to meet them “easily”, though 33.3% reported “some difficulties” and 7.7% faced “many difficulties”. The majority were Italian citizens (92.7%), with a small fraction being non-Italian (4.7%) or holding dual citizenship (2.6%). A comprehensive overview of the demographic, educational, financial, and citizenship status of the study participants by different target groups is shown in Table 1.

### 3.2. Worry About Getting HPV Infection

Out of 229 participants, the majority (41.5%) were “quite worried” about contracting HPV, with MSM (48.7%, n = 39) expressing the highest concern, followed by women under 30 (39.4%, n = 13) and PLHIV (40.0%, n = 6). Only 13.1% of respondents were “not worried”, with a higher proportion in the non-target group (16.8%, n = 18) compared to other groups (Table 2).

### 3.3. Perception of the Safety of HPV Vaccine

Among 201 respondents, a significant majority perceived the HPV vaccine as “quite safe” (55.7%) or “very safe” (35.8%). Women under 30 (96.7%, n = 29) and transgender individuals (100%, n = 6) reported the highest safety perceptions. Conversely, PLHIV showed more hesitancy, with 28.6% (n = 4) perceiving the vaccine as “quite unsafe” or “very unsafe” (Table 2).

### 3.4. Perception of Accessibility to Healthcare Facilities for HPV Vaccine

Regarding the ease of accessing healthcare facilities to obtain an HPV vaccine (n = 211), a majority found it “quite difficult” (57.8%, n = 122), especially among women under 30 (62.5%) and PLHIV (64.3%). Additionally, 23.2% (n = 49) found it “very difficult”, while only 7.1% (n = 15) found it “very easy” to access (Table 2).

### 3.5. Perception of the Affordability of HPV Vaccines

Affordability perceptions varied among the 205 respondents, with 50.2% (n = 103) finding the vaccine “quite affordable” and 25.4% deeming it “very affordable”. PLHIV (53.8%, n = 7) perceived the vaccine as the most affordable, while transgender individuals reported the highest perception of it being “not at all affordable” (33.3%, n = 2) (Table 2).

### 3.6. Knowledge of Vaccine Campaign Targets in ER

Out of 228 respondents, 63.2% (n = 144) were aware of vaccine campaign targets in the Emilia-Romagna (ER) region, with the highest awareness among women under 30 (65.6%) and the lowest among transgender individuals (33.3%) (Table 2).

### 3.7. Vaccine Uptake

The highest uptake was reported among women under 30 (72.7%) and PLHIV (60.0%). MSM had a lower uptake (41.3%), and the group not specifically targeted obviously had the lowest uptake (15.6%). Overall, a significant proportion of STIs clinic users had not received the vaccine (54.6%, n = 126). A small fraction (12.1%, n = 28) was unsure or did not remember if they had been vaccinated (Table 2).

### 3.8. Multivariate Analysis

In the multivariate analysis, age was inversely associated with worry about getting the HPV infection (OR: 0.94, 95% CI: 0.91–0.98) but showed no significant associations with perceptions of vaccine safety, access, knowledge of campaign targets, or vaccine uptake. Women under 30 years old had a significantly higher vaccine uptake (OR: 164.0, 95% CI: 17.2–1560.18) compared to STIs clinic users who were not part of this specific HPV vaccine campaign target, despite showing no significant associations with other factors. MSM demonstrated significantly higher vaccine uptake (OR: 3.53, 95% CI: 1.37–9.11) and significantly lower knowledge of vaccine campaign targets (OR: 0.33, 95% CI: 0.15–0.70). PLHIV were more likely to worry about the infection (OR: 3.50, 95% CI: 0.81–15.10), although these findings were not significant, and perceived the vaccine as less safe (OR: 0.16, 95% CI: 0.03–0.83) (Table 3).

## 4. Discussion

This cross-sectional study aimed to explore HPV vaccine uptake and awareness among STIs clinic users, targeting categories eligible for free vaccination. The survey aimed to intercept hard-to-reach communities such as MSM, PLHIV, and transgender people, to whom the offer has only recently been extended in Emilia-Romagna, Italy.

Certain facilitators and barriers to vaccination seem to be more relevant and impactful for SGM [20]. Investigating these social and behavioral factors associated with vaccine refusal or delay could support local health authorities to implement targeted interventions aimed at improving HPV vaccine coverage and contribute to the reduction in HPV-related health disparities among vulnerable populations.

The HPV vaccine uptake differed across the sample categories, with the lowest uptake observed in the population not targeted for the free vaccine offer and the highest uptake among women under 30 years of age (72.7%). The data indicated a generally low perception of risk associated with HPV infection, with nearly half of the sample reporting that they were either only slightly worried or not worried at all about the infection. Additionally, over 30 percent of all target categories, including a significant portion of PLHIV, were unaware of the specific HPV vaccination campaign. Multivariate analysis revealed that being a woman under 30 (OR = 164.0, 95% CI: 17.2–1560.18) or MSM (OR = 3.53, 95% CI: 1.37–9.11) statistically significantly increased the likelihood of having received the HPV vaccine and showed a significantly inverse association between age and the concern of contracting the infection.

Taking into account the historical target of the vaccination campaign, it is not surprising that the most consistent adherence in our sample was observed among women under 30. Our data align with the coverage rates reported by the Ministry of Health [21], suggesting strong receptivity among this group. Although, as also confirmed by a recent cross-sectional study conducted in Italy, the overall coverage remains suboptimal compared to the 95% threshold at 15 years old recommended by the National Vaccination Prevention Plan [22].

Multivariate analysis disclosed that targeted efforts to reach MSM might have been somehow effective, as this subgroup also demonstrated a higher likelihood of having received the HPV vaccine if compared to those not in this population subgroup. Despite this, more than half of the MSM population in our sample had not yet received the vaccine. Our results are just slightly more encouraging than those reported in a 2021 meta-analysis, which indicate an international average uptake rate among MSM of 37% across all studies, along with vaccination completion rates—not included in our analysis—of 28% [23]. Further research as well as implemented tailored strategies seem to be needed to fill the gap between individual decision-making processes and vaccination, given the still relatively low coverage rates among this category. For example, a randomized controlled study conducted in the United States in 2021 evaluated the effectiveness of a text-message-based HPV vaccination intervention for young MSM and showed encouraging results. In the study, the HPV vaccine uptake was significantly higher among the intervention group compared to the control group. This approach, although challenging to implement in the Italian context due to the lack of contact information for individuals belonging to SGM, suggests that targeted public health activities aimed at increasing awareness among specific population groups can be very effective.

Notably, a not-negligible portion of our sample failed to perceive the threat, reporting being “not worried” or only “a little bit worried” about contracting the infection. Similar findings are confirmed in the literature and seem to represent a barrier to vaccination [18,24]. The lack of risk perception among our sample could be a consequence of early Public Health strategies, which focused on female-oriented HPV vaccination as protection against cervical cancer [25], neglecting other health risks such as genital warts and anal and oropharyngeal cancers.

This evidence highlights the necessity of launching information campaigns that address the various risks associated with HPV infection. Interventions grounded in the information–motivation–behavioral skills model, which posits that information, motivation, and behavioral skills are crucial for fostering and sustaining health behavior changes, may prove particularly effective in this context. For instance, a Chinese randomized controlled trial found that the rates of willingness to undergo HPV vaccination were significantly higher in the intervention group compared to the control groups, with the intervention group showing a notable increase in willingness from baseline to post-intervention [26].

Regarding vaccine safety perception, PLHIV showed a significant degree of hesitancy, while most participants assessed the vaccine as “quite safe”. To improve prevention strategies, it is essential to address users’ concerns about vaccine safety, as they might lead to vaccine refusal [22]. Our findings suggest it is crucial to prioritize and reassure highly vulnerable populations such as PLHIV, encouraging them to receive the HPV vaccination. Several meta-analyses highlight the significant disease burden associated with HIV/HPV co-infection [11,27].

Another major issue highlighted by this study is a substantial proportion of individuals unaware of being targets of the vaccination campaign. PLHIV and transgender individuals represented only a small portion of our sample. However, two-thirds of PLHIV and transgender individuals who filled in the questionnaire were unaware that they could receive the vaccination free of charge. These findings underscore the need for health literacy campaigns that not only increase knowledge of the benefits of vaccination itself, but also raise awareness of eligibility for free vaccination. Communication efforts especially need to be directed toward transgender individuals, since they have only recently been introduced into the vaccine program in Emilia-Romagna, Italy. Moreover, as recent studies have pointed out, they may experience unpreparedness by health care personnel and may have doubts about the vaccination’s appropriateness in relation to their gender identity [20,28,29].

A recommendation from a healthcare provider appears to be a strong predictor for obtaining the HPV vaccination [30]. Several recent studies emphasize the importance of patient engagement in primary and sexual health care to facilitate completion of the HPV vaccine series [15,31,32]. Therefore, greater involvement of STI clinics in providing HPV vaccination consultations and recommendations could be valuable. This involvement would not only help in disseminating information about accessing vaccination and increasing risk perception awareness but also in raising awareness among target populations about the availability of free vaccination services, especially for those belonging to SGM.

### Limitations

The current study has several limitations that should be acknowledged. Firstly, due to its cross-sectional design, causal relationships cannot be inferred from the findings. Additionally, caution is advised when generalizing the results, as the study focused on a small convenience sample, which may not be representative of the broader population. Furthermore, even among participants who self-reported HPV vaccination, verification of complete vaccination cycles was not feasible. Finally, the study may also be susceptible to selection bias, given that sensitive information, such as gender identity and sexual orientation, was requested through self-disclosure. For this reason, it was emphasized multiple times in the questionnaire and by the operators that the survey was completely anonymous.

Despite these limitations and acknowledging efforts made to address them, conducting our study at one of Italy’s leading STI centers—characterized by a high daily patient flow—enabled us to effectively identify the target population for HPV vaccination outreach. Furthermore, conducting the survey across all operating days and hours over a week allowed us to capture a representative sample of the center’s attendees. This study also includes data on minority populations, which are often challenging to reach, providing initial insights into HPV vaccination among SGM individuals.

## 5. Conclusions

The study offers valuable insights into HPV vaccination uptake and awareness among under-researched minority groups, revealing lower vaccination rates among SGM attending the STI clinic in Bologna compared to the general HPV vaccination target population. By examining social and behavioral factors associated with non-vaccination—the foremost among them being the lack of awareness of eligibility for HPV vaccination—the study provides critical information to strengthen public health campaigns in the local context. The findings underscore not only the importance of increasing awareness of free vaccination eligibility among target populations (including young women, men who have sex with men, people living with HIV, and transgender individuals) but also the need for informational campaigns to emphasize risks associated with HPV infection and vaccine safety. Finally, involving STI clinics in promoting these public health campaigns may prove to be an effective strategy to enhance awareness and uptake within these communities.

## Figures and Tables

**Table 1:** Main features of the sample by HPV vaccine campaign targets in Emilia-Romagna Region, Italy (n = 236) ^a^.

		Women ≤ 30 yo ^b^	MSM ^c^	PLHIV ^d^	Transgender	Not Target	Total
n (%)		33 (14.0)	80 (33.9)	16 ^e^ (6.8)	7 (3.0)	110 (46.6)	236 (100.0)
Age (n = 196)		25.0 ± 2.5	36.3 ± 10.1	44.6 ± 9.2	34.0 ± 17.3	37.2 ± 16.7	35.0 ± 11.8
Educational level (n = 236)	No education	0 (0.0)	0 (0.0)	1 (6.2)	0 (0.0)	1 (0.9)	2 (0.9)
	Primary education/Middle School	2 (6.1)	5 (6.2)	3 (18.8)	3 (42.9)	8 (7.3)	19 (8.1)
	High School	15 (45.4)	27 (33.8)	4 (25.0)	3 (42.9)	35 (31.8)	81 (34.3)
	Bachelor’s degree	16 (48.5)	41 (51.3)	7 (43.8)	1 (14.2)	55 (50.0)	116 (49.1)
	Higher Education/PhD	0 (0.0)	7 (8.7)	1 (6.2)	0 (0.0)	11 (10.0)	18 (7.6)
With your financial resources can you meet your needs? (n = 234)	Very easily	2 (6.1)	17 (21.2)	6 (37.5)	1 (16.7)	21 (19.1)	42 (18.0)
	Easily	16 (48.5)	30 (37.5)	5 (31.2)	3 (50.0)	45 (40.9)	96 (41.0)
	With some difficulties	11 (33.3)	26 (32.5)	3 (18.8)	1 (16.7)	37 (33.6)	78 (33.3)
	With many difficulties	4 (12.1)	7 (8.8)	2 (12.5)	1 (16.6)	7 (6.4)	18 (7.7)
Citizenship (n = 232)	Italian	31 (96.9)	75 (94.9)	16 (100.0)	4 (67.6)	100 (90.9)	215 (92.7)
	Non-Italian	0 (0.0)	4 (5.1)	0 (0.0)	1 (16.7)	6 (5.5)	11 (4.7)
	Dual	1 (3.1)	0 (0.0)	0 (100.0)	1 (16.7)	4 (3.6)	6 (2.6)

^a^ One man born in 2006, part of the sample, was the only one belonging to his target category and was therefore not shown. ^b^ The abbreviation “yo” stands for “years old”. ^c^ “Men who have sex with men”. ^d^ “People living with HIV”. ^e^ Of the 16 PLHIV, 11 were also MSM.

**Table 2:** Information about HPV vaccination among STIs clinic users by HPV vaccine campaign targets in Emilia-Romagna Region, Italy.

		Women ≤ 30 yo ^a^	MSM ^b^	PLHIV ^c^	Transgender	Not Target	Total
n (%)							
Worry about getting HPV infection (n = 229)	Not worried	3 (9.1)	9 (11.2)	1 (6.7)	0 (0.0)	18 (16.8)	30 (13.1)
	A little worried	10 (30.3)	25 (31.3)	4 (26.7)	2 (40.0)	38 (35.5)	77 (33.6)
	Quite worried	13 (39.4)	39 (48.7)	6 (40.0)	2 (40.0)	40 (37.4)	95 (41.5)
	Very worried	7 (21.2)	7 (8.8)	4 (26.6)	1 (20.0)	11 (10.3)	27 (11.8)
Perception of the safety of HPV vaccine (n = 201)	Very unsafe	1 (3.3)	2 (2.9)	2 (14.3)	0 (0.0)	1 (1.1)	6 (3.0)
	Quite unsafe	0 (0.0)	5 (7.1)	2 (14.3)	0 (0.0)	6 (6.7)	11 (5.5)
	Quite safe	15 (50.0)	33 (47.1)	8 (57.1)	5 (83.3)	58 (64.4)	112 (55.7)
	Very safe	14 (46.7)	30 (42.9)	2 (14.3)	1 (16.7)	25 (27.8)	72 (35.8)
Perception of how easy it is to access healthcare facilities to obtain an HPV vaccine (n = 211)	Very difficult	0 (0.0)	6 (8.1)	0 (0.0)	1 (16.7)	7 (7.5)	15 (7.1)
	Quite difficult	2 (6.2)	11 (14.9)	1 (7.1)	1 (16.7)	12 (12.9)	25 (11.9)
	Quite easy	20 (62.5)	41 (55.4)	9 (64.3)	4 (66.6)	55 (59.1)	122 (57.8)
	Very easy	10 (31.3)	16 (21.6)	4 (28.6)	0 (0.0)	19 (20.4)	49 (23.2)
Perception of the affordability of HPV vaccines (n = 205)	Not at all affordable	1 (3.1)	4 (5.6)	2 (15.4)	2 (33.3)	8 (8.9)	16 (7.8)
	Little affordable	3 (9.4)	9 (12.5)	0 (0.0)	1 (16.7)	22 (24.4)	34 (16.6)
	Quite affordable	18 (56.2)	35 (48.6)	4 (30.8)	3 (50.0)	46 (51.1)	103 (50.2)
	Very affordable	10 (31.3)	24 (33.3)	7 (53.8)	0 (0.0)	14 (15.6)	52 (25.4)
Knowledge of vaccine campaign targets in ER (n = 228)	Yes	21 (65.6)	37 (47.4)	5 (35.7)	2 (33.3)	82 (76.6)	144 (63.2)
	No	11 (34.4)	41 (52.6)	9 (64.3)	4 (66.7)	25 (23.4)	84 (36.8)
Vaccine uptake	Yes	24 (72.7)	33 (41.3)	9 (60.0)	3 (60.0)	17 (15.6)	177 (33.3)
	No	7 (21.2)	43 (53.7)	4 (26.7)	1 (20.0)	73 (67.0)	126 (54.6)
	Do not know/remember	2 (6.1)	4 (5.0)	2 (13.3)	1 (20.0)	19 (17.4)	28 (12.1)

^a^ The abbreviation “yo” stands for “years old”. ^b^ “Men who have sex with men”. ^c^ “People living with HIV”.

**Table 3:** Multivariate analysis identifying HPV vaccine campaign target populations associated with “worry about getting the HPV infection”, “perception of the HPV vaccine as safe”, “perception of the access to get an HPV vaccine as easy”, “Knowledge of vaccine campaign targets in ER”, and HPV vaccine uptake.

	Worry About Getting the HPV Infection	Perception of the HPV Vaccine as Safe	Perception of the Access to Obtain an HPV Vaccine as Easy	Knowledge of Vaccine Campaign Targets in ER	Vaccine Uptake
Age	0.94 (0.91–0.98) *	1.02 (0.95–1.10)	1.05 (0.99–1.10)	0.99 (0.96–1.02)	1.03 (0.99–1.87)
Women ≤ 30 yo	0.77 (0.26–2.27)	1.66 (0.16–17.60)	7.67 (0.88–66.74)	0.43 (0.14–1.37)	164.0 (17.2–1560.18) *
MSM	1.47 (0.70–3.12)	0.99 (0.26–3.81)	0.77 (0.31–1.92)	0.33 (0.15–0.70) *	3.53 (1.37–9.11) *
PLHIV	3.50 (0.81–15.10)	0.16 (0.03–0.83) *	2.29 (0.26–19.94)	0.40 (0.10–1.59)	3.62 (0.74–17.63)
Transgender	1.34 (0.13–13.98)	1	1.10 (0.10–12.48)	0.89 (0.10–7.89)	9.10 (0.70–118.04)

* in bold the variables found to be statistically significant in the multivariate analysis performed.

## Data Availability

The data presented in this study are available on request from the corresponding author.

## Supplementary Materials

The following supporting information can be downloaded at https://www.mdpi.com/article/10.3390/ijerph21111515/s1, File S1: Questionnaire Instrument
