# Supplementary material for: HPV Vaccine Awareness and Uptake Among Sexually Transmitted Infections Clinic Users: A Cross-Sectional Study in Bologna, Italy
Source: Int J Environ Res Public Health. 2024 Nov 14;21(11):1515. doi: 10.3390/ijerph21111515 (PMC11593965; doi:10.3390/ijerph21111515)
Supplement: Supplementary file 1 [file ijerph-21-01515-s001.zip › ijerph-3150130-supplementary.pdf]

Supplementary Material

STROBE Checklist

STROBE Statement—Checklist of items that should be included in reports of ***cross-sectional studies***

	Item No	Recommendation
**Title and abstract**	1	(*a*) Indicate the study's design with a commonly used term in the title or the abstract
		(*b*) Provide in the abstract an informative and balanced summary of what was done and what was found
**Introduction**		
Background/rationale	2	Explain the scientific background and rationale for the investigation being reported
Objectives	2	State specific objectives, including any prespecified hypotheses
**Methods**		
Study design	2	Present key elements of study design early in the paper
Setting	2,3	Describe the setting, locations, and relevant dates, including periods of recruitment, exposure, follow-up, and data collection
Participants	2,3	(*a*) Give the eligibility criteria, and the sources and methods of selection of participants
Variables	3	Clearly define all outcomes, exposures, predictors, potential confounders, and effect modifiers. Give diagnostic criteria, if applicable
Data sources/ measurement	3	For each variable of interest, give sources of data and details of methods of assessment (measurement). Describe comparability of assessment methods if there is more than one group
Bias	3	Describe any efforts to address potential sources of bias
Study size	3	Explain how the study size was arrived at
Quantitative variables	3	Explain how quantitative variables were handled in the analyses. If applicable, describe which groupings were chosen and why
Statistical methods	3,4	(*a*) Describe all statistical methods, including those used to control for confounding
		(*b*) Describe any methods used to examine subgroups and interactions
		(*c*) Explain how missing data were addressed
		(*d*) If applicable, describe analytical methods taking account of sampling strategy
		(*e*) Describe any sensitivity analyses
**Results**		

Participants	4	(a) Report numbers of individuals at each stage of study—eg numbers potentially eligible, examined for eligibility, confirmed eligible, included in the study, completing follow-up, and analysed
		(b) Give reasons for non-participation at each stage
		(c) Consider use of a flow diagram
Descriptive data	4	(a) Give characteristics of study participants (eg demographic, clinical, social) and information on exposures and potential confounders
		(b) Indicate number of participants with missing data for each variable of interest
Outcome data	5	Report numbers of outcome events or summary measures
Main results	5,6	(*a*) Give unadjusted estimates and, if applicable, confounder-adjusted estimates and their precision (eg, 95% confidence interval). Make clear which confounders were adjusted for and why they were included
		(*b*) Report category boundaries when continuous variables were categorized
		(*c*) If relevant, consider translating estimates of relative risk into absolute risk for a meaningful time period
Other analyses	6,7	Report other analyses done—eg analyses of subgroups and interactions, and sensitivity analyses
**Discussion**		
Key results	7,8	Summarise key results with reference to study objectives
Limitations	9	Discuss limitations of the study, taking into account sources of potential bias or imprecision. Discuss both direction and magnitude of any potential bias
Interpretation	7,8	Give a cautious overall interpretation of results considering objectives, limitations, multiplicity of analyses, results from similar studies, and other relevant evidence
Generalisability	9	Discuss the generalisability (external validity) of the study results
**Other information**		
Funding	9	Give the source of funding and the role of the funders for the present study and, if applicable, for the original study on which the present article is based

*Give information separately for exposed and unexposed groups.

Note: An Explanation and Elaboration article discusses each checklist item and gives methodological background and published examples of transparent reporting. The STROBE checklist is best used in conjunction with this article (freely available on the Web sites of PLoS Medicine at http://www.plosmedicine.org/, Annals of Internal Medicine at http://www.annals.org/, and Epidemiology at http://www.epidem.com/). Information on the STROBE Initiative is available at www.strobe-statement.org.

English version of the questionnaire instrument

HPV Vaccination: "What do (not) you know?"

Socio-demographic questions

1.Year of birth

____

2.What is the sex you were assigned at birth, as indicated on your birth certificate?
○ Male
○ Female

3.How do you currently describe yourself (select all options that may apply)?
○ Female
○ Male
○ Transgender
○ Other:

4. Education level:
○ No degree
○ Elementary/middle school diploma.
○ High school diploma
○Bachelor's/master's degree
○ Doctoral/post-doctoral degree

5.Which of the following options best represents how you identify yourself:
○ Heterosexual
○ Bisexual
○ Homosexual
○ Don't know
○ Other: ______________

6.What is your nationality?
○Italian
○Foreign
○Double (Italian and Foreign)

7.With the financial resources available (from your own or family income) how do you make ends meet?
○Very easily
○Quite easily
○With some difficulty
○With a lot of difficulty

Health related questions and HPV-related questions

8. Have you ever been tested for HIV?
○ Yes
○ No
○ I prefer not to declare it

9.If you answered yes to the previous question, what was the result of the last HIV test you performed?
○ Positive
○Negative
○I prefer not to declare it

10.How concerned are you about contracting papillomavirus (HPV) infection?
○ Very little ○ A little ○ A fair amount ○ A lot

11.Have you already had the HPV vaccine?
○Yes
○No
○ Don't know/don't remember

12.If you have already done it, did you receive the HPV vaccine for free or did you pay to get it?
○Free of charge
○Payment
○ Don't know/don't remember

13.Would you get the HPV vaccine?
○Yes
○No
○Don't know

14. How safe do you think the HPV vaccine is?
○ Very little ○Slightly ○Somewhat ○ Very much

15. How easy do you think it is for you to access facilities to receive the vaccine for HPV?
○ Very little ○Slightly ○Somewhat ○ Very much

16. How affordable do you think HPV vaccination is?
○ Very little ○ Slightly ○ Somewhat ○ Very much

17.Did you know that the Emilia Romagna region recommends and offers free up to age 45 the vaccination for HPV to MSM (men who have sex with men), PLHIV (people living with HIV), transgender people, and people who engage in sex work?
○Yes
○No
